# Metabarcoding data analysis revealed the plant dietary variation of long-tailed macaque *Macacafascicularis* (Cercopithecidae, Cercopithecinae) living in disturbed habitats in Peninsular Malaysia

**DOI:** 10.3897/BDJ.10.e89617

**Published:** 2022-09-27

**Authors:** Nur Azimah Osman, Muhammad Abu Bakar Abdul-Latiff, Abd Rahman Mohd-Ridwan, Salmah Yaakop, Kayal Vizi Karuppannan, Badrul Munir Md-Zain

**Affiliations:** 1 Department of Biological Sciences and Biotechnology, Faculty of Science and Technology, Universiti Kebangsaan Malaysia, 43000 Bangi, Selangor, Malaysia Department of Biological Sciences and Biotechnology, Faculty of Science and Technology, Universiti Kebangsaan Malaysia 43000 Bangi, Selangor Malaysia; 2 School of Biology, Faculty of Applied Sciences, Universiti Teknologi Mara Negeri Sembilan, 72000 Kuala Pilah, Negeri Sembilan, Malaysia School of Biology, Faculty of Applied Sciences, Universiti Teknologi Mara Negeri Sembilan 72000 Kuala Pilah, Negeri Sembilan Malaysia; 3 Special Interest Group (ECONATREA), School of Biology, Faculty of Applied Sciences, Universiti Teknologi Mara Negeri Sembilan, 72000 Kuala Pilah, Negeri Sembilan, Malaysia Special Interest Group (ECONATREA), School of Biology, Faculty of Applied Sciences, Universiti Teknologi Mara Negeri Sembilan 72000 Kuala Pilah, Negeri Sembilan Malaysia; 4 Faculty of Applied Sciences and Technology, Universiti Tun Hussein Onn Malaysia (Pagoh Campus), 84000, Muar, Johor, Malaysia Faculty of Applied Sciences and Technology, Universiti Tun Hussein Onn Malaysia (Pagoh Campus) 84000, Muar, Johor Malaysia; 5 Oasis Integrated Group (OIG), Institute for Integrated Engineering (I²E), Universiti Tun Hussein Onn Malaysia, 86400 Parit Raja, Johor, Malaysia Oasis Integrated Group (OIG), Institute for Integrated Engineering (I²E), Universiti Tun Hussein Onn Malaysia 86400 Parit Raja, Johor Malaysia; 6 Centre for Pre-University Studies, Universiti Malaysia Sarawak, 94300 Kota Samarahan, Sarawak, Malaysia Centre for Pre-University Studies, Universiti Malaysia Sarawak 94300 Kota Samarahan, Sarawak Malaysia; 7 Department of Wildlife and National Parks (DWNP) KM10, Jalan Cheras, 56100 Kuala Lumpur, Malaysia Department of Wildlife and National Parks (DWNP) KM10, Jalan Cheras 56100 Kuala Lumpur Malaysia

**Keywords:** Malaysian primates, Southeast Asia, DNA metabarcoding, *trn*L, next-generation sequencing

## Abstract

The long-tailed macaque (*Macacafascicularis*) has a wide range in both Peninsular Malaysia and Borneo. Although the primates are especially vulnerable to habitat alterations, this primate lives in disturbed habitats due to human-induced land-use. Thus, this study presents a faecal metabarcoding approach to clarify the plant diet of long-tailed macaques from five locations in Peninsular Malaysia to represent fragmented forest, forest edge, island and recreational park habitats. We extracted genomic DNA from 53 long-tailed macaque faecal samples. We found 47 orders, 126 families, 609 genera and 818 species across these five localities. A total of 113 plant families were consumed by long-tailed macaques in Universiti Kebangsaan Malaysia, 61 in the Malaysia Genome and Vaccine Institute, 33 in Langkawi Island, 53 in Redang Island and 44 in the Cenderawasih Cave. Moraceae (33.24%) and Fabaceae (13.63%) were the most common families consumed by long-tailed macaques from the study localities. We found that habitat type impacted diet composition, indicating the flexibility of foraging activities. This research findings provide an understanding of plant dietary diversity and the adaptability of this macaque with the current alteration level that applies to long-tailed macaque conservation management interest in the future.

## Introduction

*Macacafascicularis* (Raffles, 1821) is a cercopithecine primate also known as long-tailed macaque, crab-eating macaque or cynomolgus macaque. The native range of this species includes most of mainland Southeast Asia, such as Malaysia, Indonesia, Singapore, Thailand, Laos, Vietnam, Cambodia, Myanmar and the Philippines (Roos et al. 2014). It is the most abundant macaque species in Malaysia with a wide range in Peninsular Malaysia and Borneo (Abdul-Latiff et al. 2014a, Abdul-Latiff et al. 2014b, Kamarul et al. 2014). However, its population is declining due to habitat loss and degradation, trapping and trade for pharmaceutical research (Eudey 2008). Recently, human-induced land-use and environmental changes have been major threats to many South and Southeast Asian primate populations in the 21^st^ century (Amano et al. 2021). As a result, this species is listed as endangered in the International Union for Conservation of Nature Red List (Hansen et al. 2022).

Long-tailed macaques live in several habitats with varying disturbance levels in Malaysia, including degraded and secondary rainforests, lowland primary rainforests, shrubland, mangroves, islands, agricultural areas, recreational parks and human settlements (Kamarul et al. 2014, Abdul-Latiff et al. 2017a, Dzulhelmi et al. 2019, Tee et al. 2019). Previous studies on long-tailed macaques in the disturbed habitats of Peninsular Malaysia included population surveys, daily behaviour and ecology (Hambali et al. 2012), with further analyses on human-macaque conflict, activity budget, phylogenetic analysis and feeding behaviour (Abdul-Latiff et al. 2014a, Abdul-Latiff et al. 2014b, Ruslin et al. 2014, Ruslin et al. 2019, Abdul-Nasir et al. 2021, Zamri and Md-Zain 2022). Living in disturbed habitats may strongly influence primate dispersal, distribution and viability (Lehman et al. 2006, Almeida-Rocha et al. 2017). In addition, new behavioural strategies may appear as species respond to habitat alteration (McLennan et al. 2017). Previous studies have reported the ability of long-tailed macaques to adapt and feed on human food as behavioural flexibility that facilitates their survival in urban habitats (Ilham et al. 2017) and recreational parks (Sha et al. 2009), which shows that this species is omnivorous. Its natural diet consists of fruit, flowers, young leaves and invertebrates (Lucas and Corlett 1991). Furthermore, their high dietary flexibility allows them to live near the forest edge and frequent the anthropogenic food resources (Ruslin et al. 2019). Therefore, understanding macaques’ adaptability to natural food resources in habitat disturbance is a promising issue for assessing the degree of their persistence.

Thus, in this study we investigated the plant food diversity of the long-tailed macaques, found in disturbed habitats through the metabarcoding technique. We performed a comprehensive fresh faecal sampling of this macaque across these habitats. Therefore, molecular methods have quantified the diet of long-tailed macaque where feeding is difficult to observe. Knowledge of the fundamental aspects of dietary diversity from various habitats can help identify priority conservation areas and effectively manage these species in the conflict area. Metabarcoding diet data may assist government authorities, the Department of Wildlife and National Parks and non-governmental organisations in improving management plans and conserving long-tailed macaque.

## Material and methods

**Study area**

**Figure 1.**
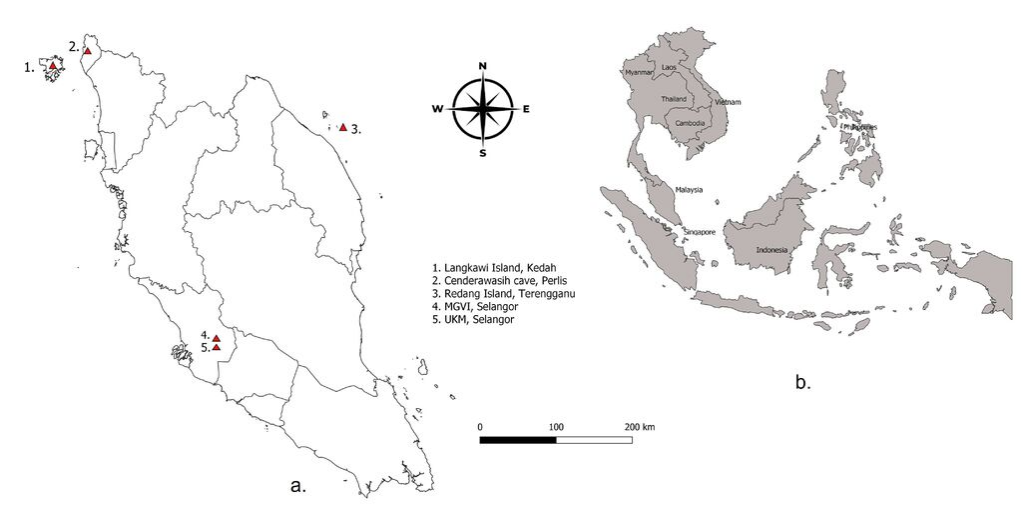
Distribution of *M.fascicularis*, a. Map of sampling locations of *M.fascicularis* in Peninsular Malaysia, b. Distribution of *M.fascicularis* in Southeast Asia.

Analysed samples in this present study were collected from non-invasive faecal material of long-tailed macaques. The samples were obtained from five localities representing four different living environments in Peninsular Malaysia: Universiti Kebangsaan Malaysia, UKM (fragmented forest); Malaysia Genome and Vaccine Institute, MGVI (fragmented forest); Langkawi Island (forest edge); Redang Island (island); and Cenderawasih Cave (recreational park) (Fig. 1). The main campus of UKM is located in Bangi, Selangor, Malaysia (2.9290° N, 101.7800° E) and is surrounded by the UKM Permanent Forest Reserve. During field observation, the samples were collected from six long-tailed macaque groups (FB1, FB2, FB3, FB4, FB5 and FB6). The MGVI is another fragmented forest area in Bangi, Selangor, Malaysia (2.9037° N, 101.7683° E). The samples (FB8) were collected in MGVI. Langkawi Island is a district and a part of the State of Kedah, Malaysia. The long-tailed macaque samples (FK9) were collected during field observation around the forest edge near Langkawi Cable Car (6.3711° N, 99.6717° E). Langkawi Cable Car is located just north of Telaga Harbour, Pantai Kok, with the entrance within “Oriental Village” at the foothills of the Mat Chincang Mountain range.

Other long-tailed macaque samples were collected around Redang Island, located in Kuala Nerus District, Terengganu, Malaysia (5.7844° N, 103.0069° E). It is one of the largest islands off the east coast of Peninsular Malaysia. Additionally, the Island is in the South China Sea approximately 24.28 nautical miles or 45 kilometres from the northern State of Terengganu (Yacob et al. 2011). The samples (FT10) were collected to represent Redang Island. Cenderawasih Cave, formerly known as Sami Cave, is a recreational park in Perlis, Malaysia (6.41426° N, 100.19285° E). It is a granite rocky hill situated at Bukit Lagi, Kangar, Perlis, Malaysia. This study site consists of a varied landscape of natural parks and recreational destination where people interact with wildlife especially primate species. This Recreational Park is adjacent to the state government administrative centre, hotel, villages and paddy field. The Park has been in operation since 2000. Samples (FR11) of long-tailed macaques were collected from this Recreational Park.

**DNA extraction**

According to the manufacturer’s protocols, the innuPREP Stool DNA Kit (Analytik Jena, Jena, Germany) extracted DNA from approximately 400 mg of long-tailed macaque faeces. First, the surface and interior of the faecal pellet were sampled for each extraction (Srivathsan et al. 2015). Then 5 µl of the extracted DNA samples of *M.fascicularis* were pooled and labelled according to the representative localities (Table 1). Next, DNA concentration was measured by spectrophotometrically using an Implen Nano Photometer. The pooled DNA concentration ranged from 1.2 to 57.3 ng/µl. Finally, the samples were stored at −20°C.

**PCR amplification**

The *trn*L intron was amplified using the previously described primers targeting the P6 loop, creating a single amplicon of approximately 90 bp (Taberlet et al. 2007). This region was amplified using the following primers: *trn*L-g forward (5´-GGGCAATCCTGAGCCAA- 3´) and *trn*L-h reverse (5´-CCATTGAGTCTCTGCACCTATC- 3´). Both primers were attached with the Illumina overhang adapter sequences forward overhang (5´-TCGTCGGCAGCGTCAGATGTGTATAAGAGACAG-3´) and reverse overhang (5´-GTCTCG TGGGCTCGGAGATGTGTATAAGAGACAG-3´). First, PCR amplification reactions were performed in a total volume of 25 µl The PCR mixture contained 12.5 µl of 2× REDTaq® ReadyMix (Sigma-Aldrich, St. Louis, Missouri, United States), 5 µl for each forward and reverse primer (20 µM) and 2.5 µl of template DNA in a final volume of 25 µl. The amplification reactions were performed in an AlphaTM PCRmax Alpha Cycler (Keison, UK) using the following programme: 95°C for 3 min, followed by 30 cycles of denaturation at 95°C for 20 s, annealing at 50°C for 15 s, extension at 72°C for 30 s and a final extension step at 72°C for 5 min.

**Illumina MiSeq-DNA sequencing of the trnL gene**

The amplicons were sent to Apical Scientific Sdn. Bhd. for next generation sequencing (NGS). Dual indices were attached to the amplicon PCR using an Illumina Nextera XT Index Kit v.2 according to the manufacturer’s protocol. The libraries’ quality was measured using the Agilent Bioanalyzer 2100 System by Agilent DNA 1000 Kit and fluorometric quantification by Helixyte GreenTM Quantifying Reagent. Finally, the libraries were normalised and pooled, based on the protocol recommended by Illumina and followed by sequencing with the MiSeq platform using 150 PE (Illumina Inc., San Diego, CA, USA).

**Statistical analysis**

All next-generation sequence data were deposited into the National Center of Biotechnology Information, under Sequence Read Archive accession numbers; SRR19576857, SRR19577193, SRR19577505, SRR19577582, SRR19577635, SRR19577594, SRR19577595, SRR19577593, SRR19577662 and SRR19577637. The quality filtering and demultiplexing of the resulting sequences were conducted using the CLC Genomic Workbench software v.12.0 (CLC) (Qiagen, Hilden, Germany) at the Genetics Laboratory in the Department of Technology and Natural Resources, Faculty of Applied Sciences and Technology, Universiti Tun Hussein Onn (UTHM), Pagoh, Johor, Malaysia. The Illumina data quality scores were initially assessed using the FASTQ file. The next step is to assign taxonomy to the reads and tally the occurrences of species. A common approach is to cluster reads at some level of similarity into representative sequences of pseudo-species called Operational Taxonomic Units (OTUs), where all reads within 97% similarity are clustered together and represented by a single OTU sequence. This approach is frequently used due to the presence of sequencing errors in the NGS reads. OTUs were aligned using the MUSCLE tool in CLC. Chimera screening and taxonomy assignment is done using the SILVA v.138 database. Rarefaction curves were plotted with the number of OTUs observed with a given sequencing depth using CLC. The alpha diversity used to assess plant species richness in the long-tailed macaque was generated using the PAST software v.4.03. The principal coordinate analysis (PCoA) using the CLC software displayed the relationship between the samples. A Venn diagram was generated to determine the shared and unique OTUs amongst the localities of these macaques at 97% similarity. A phylogenetic dendrogram and heatmap were constructed with 1,000 bootstraps following the Bray-Curtis distance to assess the relationship of the plant species community amongst these samples. Statistical significance was set at p < 0.05.

## Data resources

Fifty-three fresh faecal samples were collected from five localities. However, because of opportunistic collection methods, we could not link the faecal samples to a specific age-sex class or known individual. In addition, samples were easily distinguishable from other primates according to the physical characteristics of the fresh faeces by size, smell, form and colour. The faecal samples were collected in sterile 45 ml tubes and fixed in 95% ethanol for long-term storage (Song et al. 2016). All analysed samples were labelled and stored at −20°C.

## Results

**Figure 2.**
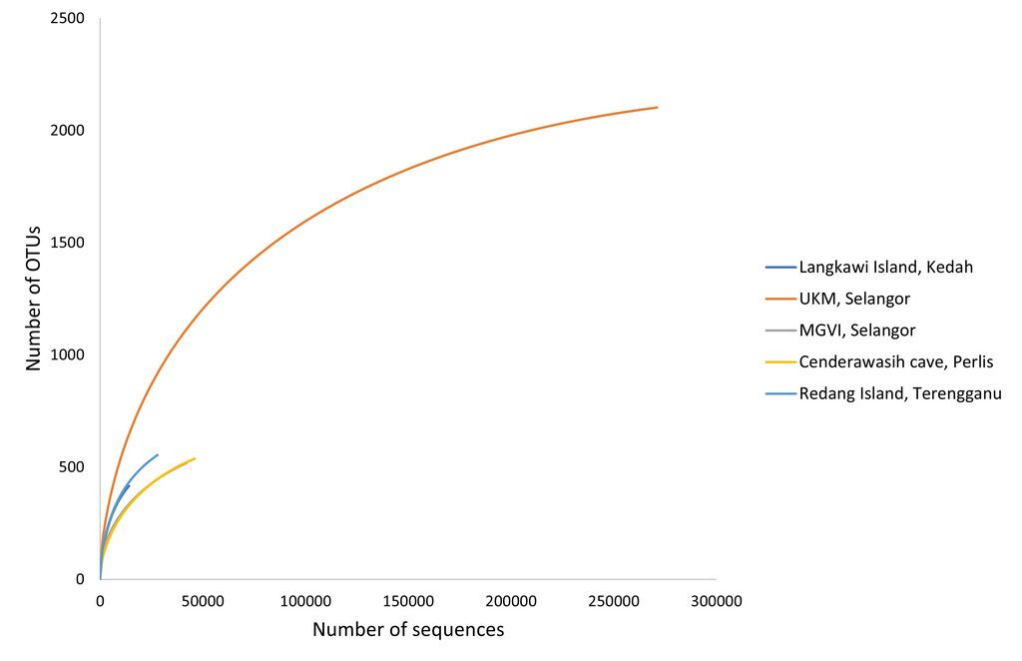
The rarefaction curve of the *trn*L gene sequence of *M.fascicularis* calculated for operational taxonomic units at similarity of 97%.

The NGS produced 595,455 reads from five pooled samples of *M.fascicularis* in different localities, ranging from 46,736 to 371,005. The final dataset obtained by sequence filtering excluded low-quality sequence reads, chimera and subsequently OTU clustering. A total of 407,354 OTU sequence reads were generated at the 97% similarity cut-off. UKM long-tailed macaque population showed the most OTU sequence reads (272,047) followed by Cenderawasih Cave (47,254), MGVI (43,157) and Redang Island (29,798), whereas the fewest reads were from Langkawi Island (15,098) (Fig. 2). The Shannon–Wiener index (H’) showed that the UKM population had the highest diversity with H’ of 3.806 and 2103 OTUs and the lowest was recorded for Langkawi Island at 2.781 and 428 OTUs (Table 2).

**Diet richness and composition of the *M.fascicularis***

**Figure 3.**
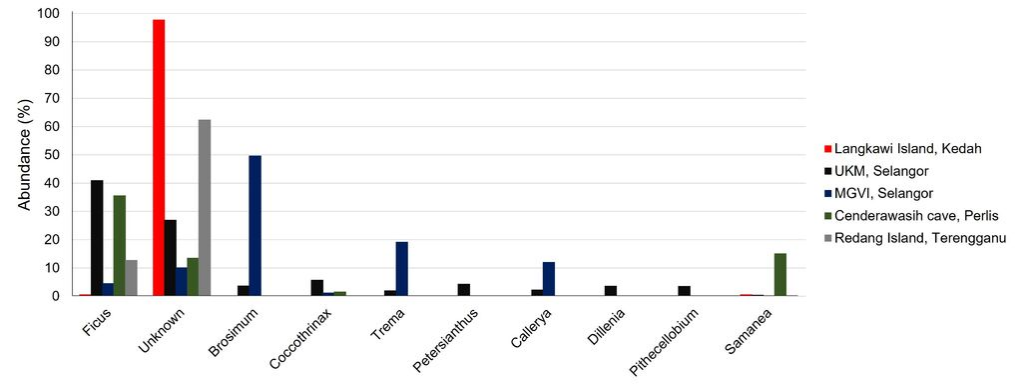
The relative abundance of *M.fascicularis* plant diet at the genus level.

The *trn*L intron sequence of the *M.fascicularis* samples was amplified. The OTUs were assigned to 47 orders, 126 families, 609 genera and 818 species across these five pool samples. A total of 113 families of plants were in UKM, MGVI (61), Langkawi Island (33), Redang Island (53) and Cenderawasih Cave samples (44). Moraceae (33.24%) and Fabaceae (13.63%) were the most common families recorded from all study localities. The unknown family was recorded at 21.22%. *Ficus* is the most abundant genus recorded in the samples from UKM (41.04%) and Cenderawasih Cave (35.68%). *Brosimum* (49.75%) is the most abundant genus in MGVI samples (Fig. 3). The non-available (N/A) (97.80% and 62.44%) were mostly recorded in Langkawi Island and Redang Island, respectively. The highest plant species consumed by *M.fascicularis* in UKM (30.13%) and Cenderawasih Cave (27.33%) were *Ficussuperba*. Conversely, *Brosimumalicastrum* (36.86%) was the most common plant consumed by this macaque in the MGVI samples. As the most abundant species is N/A, *Veprissimplicifolia* (9.24%) and *Miliciaregia* (19.99%) were recorded as the common plant species consumed by *M.fascicularis* in Langkawi Island and Redang Island samples, respectively.

**Figure 4.**
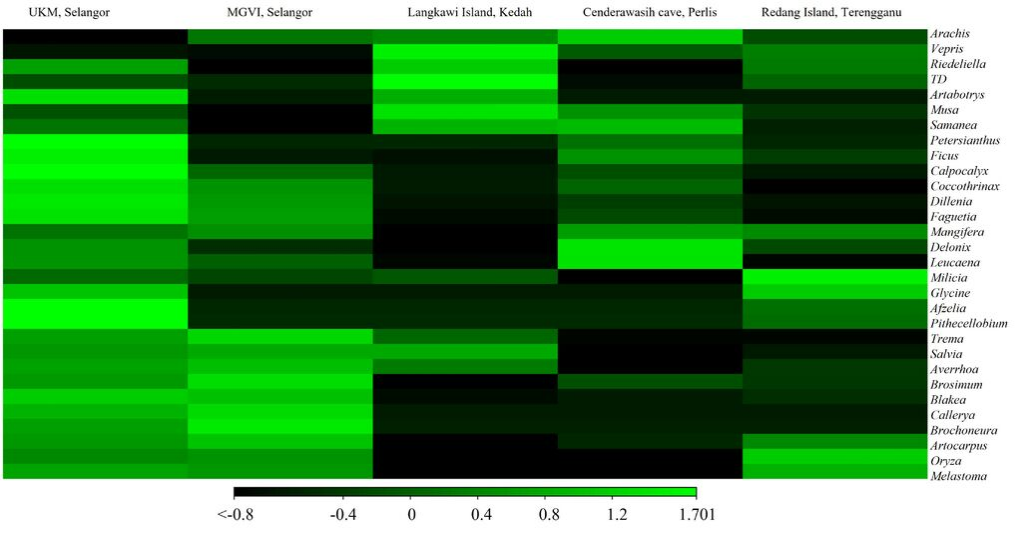
Heatmap with dendrogram at the genus level using a gradient heatmap (over 1% of the plant diversity).

Heatmap analysis revealed significant interindividual variability in the plant communities’ consumption in composition level at the five localities foraged by *M.fascicularis*. The 30 most abundant genera were adopted in the hierarchical clustering using weighted pair clustering based on Bray-Curtis measurements to evaluate the relationships between this macaque (Fig. 4). The heatmap showed the value of genera consumed by long-tailed macaque by colour and the lighter the green, the more predominant the genus consumed.

**Figure 5.**
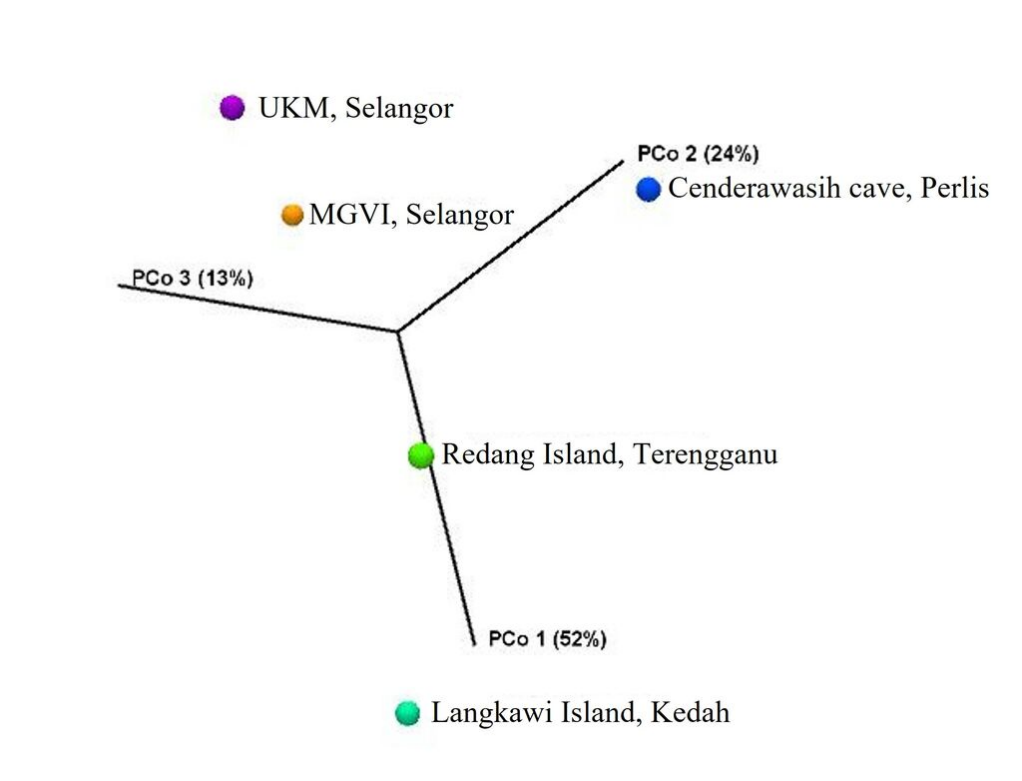
A three-dimensional plot of weighted UniFrac-based principal coordinates analysis (PCoA). The plot is created using the pairwise weighted UniFrac distances (PCo1 variability at 52%, PCo2 variability at 24% and PCo3 variability at 13%).

Beta-diversity on UniFrac-based principal coordinate analysis (PCoA) showed a dietary relationship amongst the five localities (Fig. 5). No significant difference appeared in the plant diversity consumed by long-tailed macaques at these localities, according to further permutational multivariate analysis of variance (PERMANOVA) analysis (p < 0.05).

**Figure 6.**
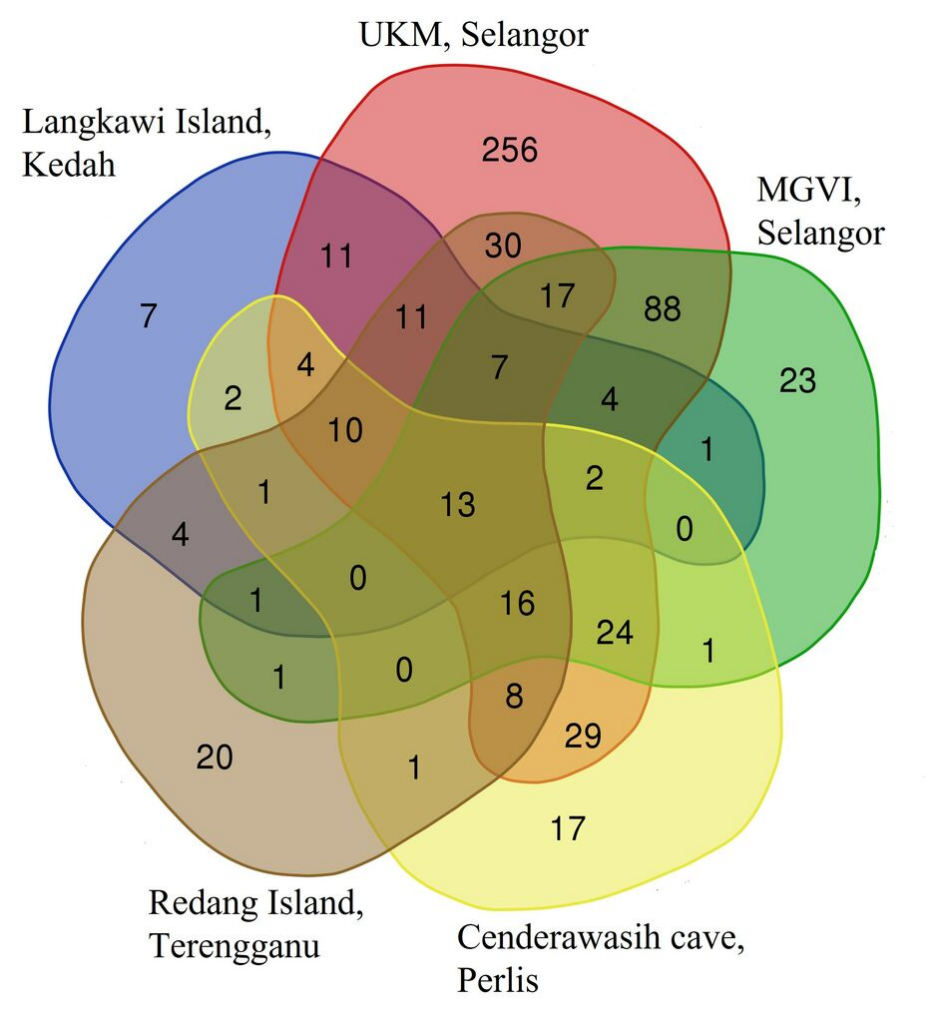
Venn diagrams summarising the number of shared plant genus amongst different locations. Coloured circles represent each location and the intersection between circles represents the number of shared plants.

Additionally, these localities had overlapping plant genera (Fig. 6). Samples from UKM and MGVI showed a larger overlap (88) than those from MGVI and Langkawi Island (1). The number of genera shared by all localities was 13.

## Discussion

This is the first study to report on the diversity of plants consumed by long-tailed macaques in disturbed habitats in Peninsular Malaysia using an advanced approach that combines DNA metabarcoding and Illumina NGS of *trn*L chloroplast genes. The data obtained from the five localities in the four different environmental settings presented a novel finding for understanding the diet of long-tailed macaques. Generally, long-tailed macaques fed on several food plants belonging to at least 693 species from 113 different families in a mixed landscape consisting of urban, agro-forested areas and forest fragments in Malaysia at UKM rather than in the other localities. These data supported the species richness of the food plants and sampling efforts performed on six macaque groups in UKM. As a lowland secondary dipterocarp fragmented forest, UKM Permanent Forest Reserve harbours important plant resources, including more than 500 species of seed plants surrounding the UKM main campus (Salleh 1999). *Ficussuperba* (deciduous fig) in the family Moraceae was the most abundant taxon detected consumed by *M.fascicularis* at UKM. Ruslin et al. (2019) identified 113 species of plants consumed by one group of long-tailed macaques at UKM who used direct feeding observations. Fig, (*Ficusvariegatae*), was one of the most consumed taxa. Similarly, long-tailed macaques at MGVI also consumed *F.superba*. Both fragmented forests, UKM and MGVI showed the larger food plant overlap consumed by long-tailed macaques. These localities shared 88 genera of plants including *Ficus*, *Brosimum* and *Coccothrinax*. Further analysis showed no significant difference in the plant diversity consumed by long-tailed macaques at these two localities. This result may be attributable to the different groups obtained at both sites and the varying distance of their home range and time spent travelling between them. Que´me´re et al. (2013) observed great shifts in dietary composition of lemur related to habitat composition and openness, suggesting high flexibility of foraging strategies. However, we did not examine the time spent feeding on different food types. Further studies should be conducted to elucidate the effect of the home range and this macaque’s feeding time. For long-tailed macaque population in the Recreational Park of Cenderawasih Cave, *F.superba* also accounted for a significantly greater proportion of the macaque’s diet. *F.superba* was the main plant diet consumed by another macaque species, *Macacaarctoides* inhabiting Perlis State Park, Wang Kelian, Perlis, Malaysia (Osman et al. 2020). Primates that live in more marginal habitats depend highly on limited critical food resources, such as figs, that play an important role in nourishing frugivores through periods of natural and imposed food shortage (Kinnaird et al. 1999, Tweheyo and Lye 2003). *M.fascicularis* is one of the primates that can be found in the forest area at Langkawi Island and Redang Island. *Veprissimplicifolia* (an evergreen shrub) is a common plant species consumed by *M.fascicularis* living in the tropical rain forest edge at Langkawi Island. The peri-urban group of *Macacasylvanus* spent one-third of its feeding activities in the shrub layer rather than in the non-urban group (Maibeche et al. 2015). *M.fascicularis* in Redang Island mostly feed on *Miliciaregia*, a tropical tree in the Moraceae family. Khairil et al. (2013) determined that Moraceae is one of the plant families in the coastal forest of Redang Island. Our findings indicated that long-tailed macaques consume varied selected plants in these habitats.

We projected that anthropogenic habitat alteration would lead to lower habitat quality for long-tailed macaques. Although the forests at UKM, MGVI, Langkawi Island and Redang Island have been minimally altered, human interaction still occurs. The disturbance level varied between the study areas. This macaque occupied a heavily altered habitat characterised by scarcity of plant food abundance and frequent human visits to the Cenderawasih Cave. However, we did not survey the availability of anthropogenic food. Thus, it is hard to verify whether this macaque highly depends on anthropogenic foods or its natural food resource. The amount of food from natural sources is consumed more by the long-tailed macaques than that from visitors at the high-altitude rainforest of Telaga Warna, West Java, Indonesia (Nila et al. 2014). Further study at this forest site showed that these macaques depend mostly on artificial food by consuming provisioned food (Julianti et al. 2020). The study group of long-tailed macaques chose their natural diet from 21 species of food plants, two species of animals, two species of insects and human food waste sources at Kuala Selangor Nature Park, Malaysia (Hambali et al. 2014). Habitat quality may also affect dietary diversity (Mohd-Daut et al. 2021, Mohd-Daut and Md-Zain 2021). Primates living in undisturbed forests with greater tree species diversity have more foraging options and are less likely to experience food scarcity than primates living in more fragmented forests (Poulsen et al. 2001, Najmuddin et al. 2019, Najmuddin et al. 2021). Intraspecific comparisons of the proportion of plant food resources in different primate groups living in habitats with varying disturbance levels are one technique for investigating how these species respond to human habitat alteration and dietary adjustments (Wieczkowski 2003, McLennan et al. 2017). The primates respond to habitat modification depending on the ecological behaviour of each species (Maibeche et al. 2015). The macaques are ecologically flexible in taking any food available in their home range and adjust their behaviour according to their abundance. Long-tailed macaques are selective feeders, but can also exploit various food sources during periods of food scarcity (Yeager 1996). Moreover, long-tailed macaques have greater ecological flexibility to adapt and interact in various habitats (Julianti et al. 2020). Forest fragmentation and habitat loss are the current threats to the population of *M.fascicularis* in Peninsular Malaysia. The populations of this primate in Malaysia migrate to human settlement areas from their natural habitat due to logging and anthropogenic activities, such as urbanisation and land-freeing for agriculture (Dzulhelmi et al. 2019). Identifying dietary variation of *M.fascicularis* is the key element that may promote the perseverance of this species in disturbed habitats. Additionally, evaluating the changes in the availability of these elements may affect their future conservation prospects.

Non-invasive fresh faecal sampling was conducted in this study without capturing, touching or restraining the long-tailed macaques (Syed-Shabthar et al. 2013, Abdul-Latiff et al. 2017b). Combining this type of sampling and the metabarcoding approach is valuable because both provide ecological and biological information without direct observation (Silva et al. 2012). Molecular methods have recently been applied to study the diet of various species where feeding is difficult to observe or quantify (Boyer et al. 2013). NGS provides a good compromise in this study as we successfully amplified the *trn*L sequences. The number of sequences obtained was higher and sufficient to evaluate the plant diet of *M.fascicularis* in various habitats rather than direct feeding observation by Hambali et al. (2014) and Ruslin et al. (2019). The chloroplast DNA, *trn*L intron has been widely evaluated as a standard sequence that provides superior performance in identifying plant species since it was designed by Taberlet et al. (1991). The outperformance of this barcoding marker has been tested in other primate species. DNA metabarcoding studies in white-faced capuchins, *Cebuscapucinus*, results in greater sequences with equal sequencing effort, greater resolution taxonomic identifications and a higher number of at least 365 plant families (Mallott et al. 2018). Furthermore, this approach also identifies plant diet in other primate species (Que´me´re et al. 2013, Srivathsan et al. 2015, Srivathsan et al. 2016, Osman et al. 2020), herbivorous birds, mammals, insects and molluscs (Valentini et al. 2008, Aziz et al. 2017, Fahimee et al. 2021). However, investigations using DNA metabarcoding are limited due to the absence of a good reference database, especially when the diet of the species being studied is not well defined. Variability in different parts of the leaf also depends on the sequence specificity of the primers; therefore, there is a potential that an unknown plant species will be undetected due to primer mispriming (Aziz et al. 2017). Notably, we used a computational approach with successive filtering steps to eliminate all sources of erroneous reads including PCR-generated chimeric sequence, primer dimmers, nuclear pseudogenes and contaminations. The quality filtering and trimming of raw reads must be performed to eliminate erroneous data before barcoding analysis since NGS may produce sequencing errors (Yang et al. 2018). We must note the lack of data on the home range and time spent feeding. However, our study provided rapid molecular plant dietary screening and documentation of *M.fascicularis* in disturbed habitats.

## Conclusions

Our study reveals the dietary variation of long-tailed macaques in disturbed habitats by *trn*L DNA metabarcoding. Using a non-invasive method allows DNA metabarcoding to reveal the diet of long-tailed macaques where it has always been difficult to obtain using direct observation. Therefore, our results propose that long-tailed macaques consume various food plants that help them survive in disturbed habitats at the edge and centre of the fragmentary and human interference areas. Furthermore, we provide data on these species’ dietary requirements and plant species availability in different habitats. Knowledge of the fundamental aspects of dietary diversity from various habitats is increasingly employed to identify priority conservation areas and effectively manage these species in the conflict area. Other food resources are needed to understand their feeding behaviour since long-tailed macaques are omnivorous. Notably, metabarcoding diet data may assist government authorities, the Department of Wildlife and non-governmental organisations in improving management plans and conservation of long-tailed macaques in the conflict area. Knowing which plants are consumed by cercopithecine primates will guide translocation processes from disturbed habitats to the undisturbed forests that harbour these important resources.

## Figures and Tables

**Table 1.** List of samples and localities of *M.fascicularis* used for plant diet analysis.

#	Pool samples code	Number of samples	Locality	Type of habitat	Coordinate
1	FB1	12	UKM, Selangor	Fragmented forest	2.9290° N, 101.7800° E
2	FB2	5	UKM, Selangor	Fragmented forest	2.9290° N, 101.7800° E
3	FB3	5	UKM, Selangor	Fragmented forest	2.9290° N, 101.7800° E
4	FB4	5	UKM, Selangor	Fragmented forest	2.9290° N, 101.7800° E
5	FB5	7	UKM, Selangor	Fragmented forest	2.9290° N, 101.7800° E
6	FB6	5	UKM, Selangor	Fragmented forest	2.9290° N, 101.7800° E
7	FB8	5	MGVI, Selangor	Fragmented forest	2.9037° N, 101.7683° E
8	FK9	3	Langkawi Island, Kedah	Forest edge	6.3711° N, 99.6717° E
9	FT10	3	Redang Island, Terengganu	Island	5.7844° N, 103.0069° E
10	FR11	3	Cenderawasih Cave, Perlis	Recreational Park	6.41426° N 100.19285° E
Total	10	53	-	-	-

**Table 2.** The number of observed operational taxonomic units, alpha diversity indices for the plant DNA from five localities of *M.fascicularis*.

Localities	Sequences	OTUs	Shannon (H')	Chao 1
UKM, Selangor	272047	2103	3.806	2198
MGVI, Selangor	43157	523	2.897	647.5
Langkawi Island, Kedah	15098	428	2.781	562.5
Cenderawasih Cave, Perlis	47254	542	2.889	650.8
Redang Island, Terengganu	29798	565	3.676	702.4
Total	**407354**	**4161**	-	-
